# Mediastinal Hibernoma on Fluorine-18 Fluorodeoxyglucose (18F-FDG) Positron-Emission Tomography/Computed Tomography (PET/CT): A Case Report and Literature Review

**DOI:** 10.7759/cureus.89553

**Published:** 2025-08-07

**Authors:** David Gutierrez Albenda, Nelson Mauricio Sánchez Hidalgo, Juan J Solano Brenes, Laura Natalia Rodríguez Varela, Julyana Murillo

**Affiliations:** 1 Cyclotron-PET/CT Laboratory, University of Costa Rica, San José, CRI; 2 School of Medicine, University of Costa Rica, San José, CRI

**Keywords:** 18f-fdg pet/ct, 18f-fluorodeoxyglucose positron-emission tomography (18f-fdg pet), brown adipose tissue, hibernoma, mediastinal hibernoma

## Abstract

Hibernomas are infrequent brown adipose tissue tumors that can mimic various other soft tissue tumors, both malignant and benign, and multiple imaging modalities are usually used in the diagnostic approach. We present the case of a 30-year-old female patient who was initially diagnosed with a mediastinal hibernoma through complete excisional biopsy in 2022 and underwent surgical resection at that time. She required a second surgical intervention in 2024 due to recurrence and is now being evaluated for a second recurrence using fluorine-18 fluorodeoxyglucose positron-emission tomography/computed tomography (18F-FDG PET/CT), reporting an extensive polylobulated mediastinal tumor intercalating normometabolic and discretely hypermetabolic zones.

Hibernomas consist of a combination of brown adipose tissue and normal adult adipose tissue. The brown adipose tissue has a higher glucose metabolism, leading to increased 18F-FDG uptake and significant radiotracer accumulation. A multidisciplinary approach is important. The use of 18F-FDG PET/CT is valuable in hibernoma evaluation, as it can detect increased metabolism compared to other tissues. This provides additional information that can complement other imaging studies. However, it is important to interpret these results carefully, as they can raise concerns for false-positive metastasis. Given the infrequent nature and somewhat difficult diagnosis, there is yet much to learn about hibernomas.

## Introduction

Hibernomas are rare benign soft tissue tumors of unknown etiology, originating from residual brown adipose tissue that persists into adult life [1]. First described in 1906, its name was coined in 1914 and comes from the resemblance to the brown fat found in hibernating animals [1-3]. Hibernomas are composed of multivacuolate adipocytes like those seen in brown fat, mixed with univacuolate adipocytes similar to those seen in typical lipomas [2,4]. Clinically, they present as a well-defined, slow-growing, painless, mobile mass that can cause symptoms by compression of nearby structures [1].

Hibernomas can pose a diagnostic challenge before pathological diagnosis as they can mimic various benign and malignant soft tissue tumors like lipoma (typical and atypical), rhabdomyosarcoma, liposarcoma, and others [1,2,4]. The most frequent localization in adults is thighs, upper extremities, trunk, and neck, but it can grow in areas in which small amounts of brown fat persist, like the mediastinum, which is not a frequent location [1,5,6]. It has a slightly higher incidence in women and a peak incidence in the third and fourth decades of life [2,5,6].

Image studies used in the diagnostic approach usually include ultrasonography (US) as the first study showing great vascularity, computerized tomography (CT) showing fat density tissue, magnetic resonance imaging (MRI) useful to differentiate from normal fat surrounding, and positron-emission tomography (PET) to evaluate functional characteristics and make differential diagnosis [1,7]. Surgical excision treatment is supposed to be curative according to the literature, as no recurrence is reported [2].

## Case presentation

A 30-year-old female patient underwent surgical intervention for superior vena cava (SVC) obstruction in 2021. She had been diagnosed with a hibernoma at the age of 28 in 2022, following a complete excisional biopsy of the mass via thoracotomy. The biopsy reported the presence of an adipose tissue tumor that was partially surrounded by a fibrous capsule, with areas of calcification. Most of the tumor consisted of immature adipose tissue featuring adipose vesicles with central nuclei, along with cytoplasmic microvesicles. There were also peripheral regions of mature adipose tissue, ischemic necrosis, and thrombi. Additionally, some myxoid peripheral zones exhibited low cellularity. The tumor tested negative for murine double minute 2 on immunohistochemical staining.

The patient underwent a second complete resection in 2024. However, the patient later presented with a mediastinal recurrence, as evidenced by a contrast-enhanced thorax CT scan, which revealed multiple atypical adenopathies in groups, most probably adipose tissue, on every mediastinal station, producing compression of the airway, along with a nodular lesion in the pleura located toward the posterior segment of the right lung's superior lobe. At that moment, the patient was symptomatic with cough and dyspnea with mild to moderate exertion. A PET/CT scan was ordered to evaluate the patient's oncological state.

A high spatial resolution PET/CT was performed using fluorine-18 fluorodeoxyglucose (18F-FDG) as a radiopharmaceutical tracer, with a reference metabolic activity of SUVmax 2.55 on the right hepatic lobe and SUVmax 1.79 on mediastinic blood pool. The 18F-FDG PET/CT revealed an extensive polylobulated mediastinal tumor with well-defined borders, measuring approximately 13.07 x 11.93 x 29 cm. The tumor exhibited heterogeneous density, primarily fat density, intercalating normometabolic and discretely hypermetabolic zones (SUVmax 2.54) and compromising cranially up to the superior limit of the cervical levels III on the right side and IV on the left side. This led to a leftward displacement of the trachea, thyroid, larynx, and esophagus, as well as a lateral displacement of the carotid arteries and internal jugular veins, which reduced the caliber of the right jugular vein. The central portion of this mass occupies the perivascular, para- and retrotracheal, subcarinal, right paraesophageal, and paracardiac spaces, displacing to the left and comprising the trachea and partially the initial segments of both principal bronchi. In addition, it laterally displaces the mediastinal vascular structures, significantly impacting the venous components. It runs distally through the preesophageal space until it reaches the diaphragmatic hiatus. Additionally, a second right subpleural tumor with similar morphometabolic characteristics is reported. An elongated hypermetabolic (SUVmax 7.09) pattern is observed along the anatomical course of the SVC. The finding is most likely inflammatory in nature and is associated with an SVC endoprosthesis.

## Discussion

Brown adipose tissue is present in newborns and regresses with age. It has thermoregulatory functions, contains higher numbers of mitochondria, and has a high rate of glucose metabolism; it may also have a potential role in adult metabolism [2,5,8]. Depots of brown adipose tissue in adults are more frequent in women than in men, and its amount is inversely correlated with the body mass index, especially in older people [8]. The body areas in which brown fat tissue persists include the scapular and interscapular regions, upper thorax and mediastinum (like the case reported in which the hibernoma is present in all mediastinal stations), thighs, shoulders, back, abdomen, retroperitoneum, intramuscular, or intraosseous [2,9]. Even though hibernomas can develop in all those places, they are statistically rare, representing only 1.6% of benign lipomatous tumors [5,7].

Macroscopically, hibernomas typically appear as yellowish-brown, well-defined, lobulated, and partially encapsulated masses with a rubbery texture. They are usually painless and grow slowly. At the time of diagnosis, hibernomas can range in size from 1 to 27 cm. In this case, the patient's hibernoma measures 29 cm on its major axis, placing it on the larger side of this spectrum. In microscopic examination, hibernomas have multivacuolar adipocytes combined with univacuolar adipocytes, associated capillary proliferation and fibrovascular septa, and at least four histologic variants have been described, including typical, mixoid, lipoma-like, and spindle cell [4,5].

**Figure 1:**
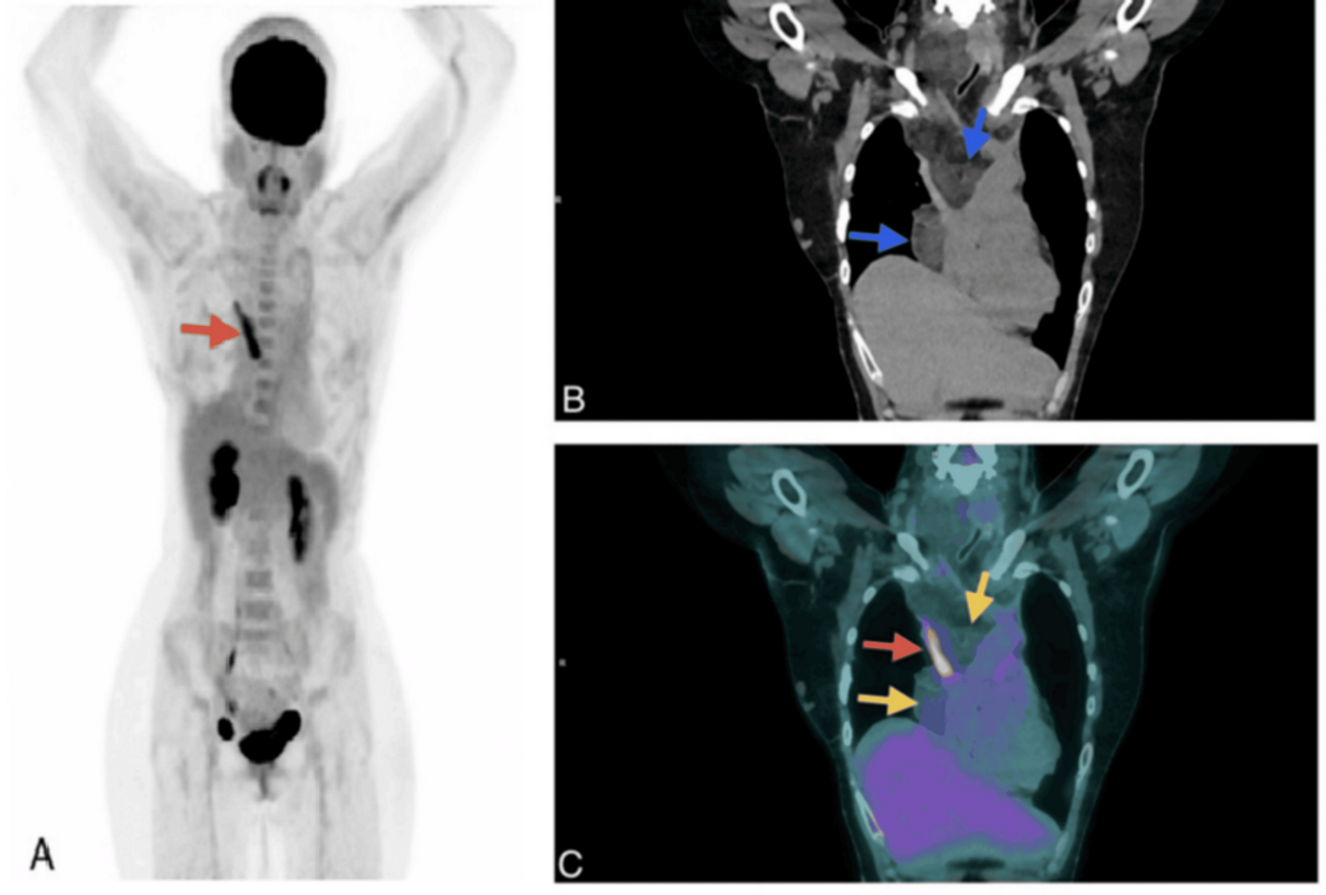
(A) FDG-PET/CT whole-body MIP showing hypermetabolism along the SVC (red arrow). (B) Coronal CT (soft-tissue window) showing a well-defined, multilobulated mediastinal mass with predominantly fat density (blue arrows). (C) Coronal PET/CT fusion showing alternating normometabolic and mildly hypermetabolic areas (yellow arrows) (SUVmax 2.54) and marked hypermetabolism (SUVmax 7.09) along the SVC (red arrow), associated with an endoprosthesis FDG-PET/CT: fluorodeoxyglucose positron-emission tomography computed tomography; MIP: maximum intensity projection; SVC: superior vena cava

**Figure 2:**
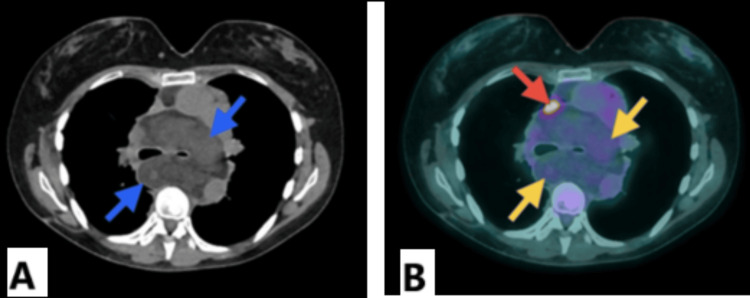
(A) Axial CT (soft-tissue window) showing a well-defined, multilobulated mediastinal mass with predominantly fat density (blue arrows). (B) Axial PET/CT fusion showing alternating normometabolic and mildly hypermetabolic areas (yellow arrows) (SUVmax 2.54) and marked hypermetabolism (SUVmax 7.09) along the SVC (red arrow), associated with an endoprosthesis CT: computed tomography; PET: positron-emission tomography; SVC: superior vena cava

In the diagnostic approach, different imaging studies, such as US, CT, MRI, and PET/CT, can be used to reflect its tissue components. CT presents hibernomas as well-defined masses with prominent adipose tissue present, and contrast-enhanced MRI may show a marked enhancement of the brown fat component of hibernomas [2,7]. On PET, the brown fat component of hibernomas shows an intense FDG accumulation because of the higher glucose metabolism mentioned before, which causes an increased FDG uptake compared to normal adult adipose tissue [2,5]. In the presented case, the CT scan reported groups of atypical adenopathies in the mediastinal stations that were not identified in the 18F-FDG PET/CT. This discrepancy likely occurred because the PET/CT interpretation focused on the overall metabolic pattern, as shown in Figures 1, 2, which illustrate the hypermetabolic areas in the mediastinum, with an SUVmax of 2.54 compared to 1.79 for the reference mediastinal blood pool. This further emphasizes the utility of 18F-FDG PET/CT as a complementary imaging study in the evaluation of hibernomas.

Differential diagnosis includes conditions such as fat tissue necrosis, atypical lipomas, rhabdomyosarcomas, and primarily liposarcomas, especially in the lower extremities. Liposarcomas also exhibit increased glucose metabolism. It is important to consider that a high 18F-FDG uptake on 18F-FDG PET/CT reflects the metabolic activity and cellular components of the mass, but not necessarily always reflects the malignant potential [1,6]. 18F-FDG PET/CT has even been used as a noninvasive method to quantify brown adipose tissue in patients undergoing scans for various diagnostic reasons, and hibernomas may also be incidental findings in patients with other pathologies undergoing this study, which may raise concerns about metastasis being a false positive [5,8,9]. Inflammatory processes are also areas of high glucose metabolism that cause high FDG uptake [6], like the reported hypermetabolism associated with the endoprosthesis of the SVC in the patient.

Definitive diagnosis must be made by histopathology, and several histologic variants mentioned before have been described, but they are of no prognostic relevance [2,10]. Election treatment is complete surgical resection. Great care should be taken to minimize bleeding risk due to the extensive vascularity of hibernomas. It is a curative procedure and has a good prognosis with no reports of recurrence, metastasis, or malignant transformation according to the literature consulted [1-3,7,10]. It is difficult to determine whether true recurrences or involuntary incomplete resections are the problem, and it is uncertain if the resections were complete, but they were reported as such. On the other hand, as already mentioned, the recurrence would be a rarity, making this case particularly interesting.

## Conclusions

It is important to adopt a multidisciplinary approach including radiology, pathology, surgery, and nuclear medicine in the assessment of hibernomas. 18F-FDG PET/CT is a useful complementary imaging modality, as it can noninvasively distinguish between normometabolic adipose tissue lesions and hibernomas by evaluating their higher glucose metabolism, it emerges as a valuable resource not only for initial evaluation but also for detecting possible recurrence, particularly in complex anatomical regions such as the mediastinum. As with any diagnostic tool it has limitations, findings must be interpreted alongside the patient’s history, clinical presentation, and other imaging studies. Clinicians should remain aware that benign lesions and inflammatory processes can also exhibit increased FDG uptake, underscoring the need for careful correlation with all available clinical and pathological data.

Our patient’s presentation fits the demographic profile reported in the literature; however, the recurrence observed in this case is unusual, as previous reports describe surgical excision as curative. This highlights the importance of continued documentation of such cases to expand current knowledge and better define the role of 18F-FDG PET/CT in the follow-up of hibernomas.
